# Single molecule sequencing and genome assembly of a clinical specimen of *Loa loa,* the causative agent of loiasis

**DOI:** 10.1186/1471-2164-15-788

**Published:** 2014-09-12

**Authors:** Luke J Tallon, Xinyue Liu, Sasisekhar Bennuru, Marcus C Chibucos, Alvaro Godinez, Sandra Ott, Xuechu Zhao, Lisa Sadzewicz, Claire M Fraser, Thomas B Nutman, Julie C Dunning Hotopp

**Affiliations:** Institute for Genome Sciences, University of Maryland School of Medicine, Baltimore, MD USA; Laboratory of Parasitic Diseases, National Institute of Allergy and Infectious Diseases, Bethesda, MD USA; Department of Microbiology and Immunology, University of Maryland School of Medicine, Baltimore, MD USA

## Abstract

**Background:**

More than 20% of the world’s population is at risk for infection by filarial nematodes and >180 million people worldwide are already infected. Along with infection comes significant morbidity that has a socioeconomic impact. The eight filarial nematodes that infect humans are *Wuchereria bancrofti, Brugia malayi, Brugia timori, Onchocerca volvulus, Loa loa, Mansonella perstans, Mansonella streptocerca*, and *Mansonella ozzardi,* of which three have published draft genome sequences*.* Since all have humans as the definitive host, standard avenues of research that rely on culturing and genetics have often not been possible. Therefore, genome sequencing provides an important window into understanding the biology of these parasites. The need for large amounts of high quality genomic DNA from homozygous, inbred lines; the availability of only short sequence reads from next-generation sequencing platforms at a reasonable expense; and the lack of random large insert libraries has limited our ability to generate high quality genome sequences for these parasites. However, the Pacific Biosciences single molecule, real-time sequencing platform holds great promise in reducing input amounts and generating sufficiently long sequences that bypass the need for large insert paired libraries.

**Results:**

Here, we report on efforts to generate a more complete genome assembly for *L. loa* using genetically heterogeneous DNA isolated from a single clinical sample and sequenced on the Pacific Biosciences platform. To obtain the best assembly, numerous assemblers and sequencing datasets were analyzed, combined, and compared. Quiver-informed trimming of an assembly of only Pacific Biosciences reads by HGAP2 was selected as the final assembly of 96.4 Mbp in 2,250 contigs. This results in ~9% more of the genome in ~85% fewer contigs from ~80% less starting material at a fraction of the cost of previous Roche 454-based sequencing efforts.

**Conclusions:**

The result is the most complete filarial nematode assembly produced thus far and demonstrates the utility of single molecule sequencing on the Pacific Biosciences platform for genetically heterogeneous metazoan genomes.

**Electronic supplementary material:**

The online version of this article (doi:10.1186/1471-2164-15-788) contains supplementary material, which is available to authorized users.

## Background

More than 180 million people are infected with vector-borne filarial nematodes worldwide with >20% of the world’s population at risk for infection. Eight species of filarial nematodes are known to infect humans including *Wuchereria bancrofti, Brugia malayi, Brugia timori, Onchocerca volvulus, Loa loa, Mansonella perstans, Mansonella streptocerca*, and *Mansonella ozzardi*. Of these eight, only the first five cause substantial pathology and have been the focus of whole genome sequencing efforts thus far [1, 2].

Humans are the definitive hosts for all of the medically-relevant filarial nematodes; only *B. malayi* naturally infects other vertebrates. Thus, for most filariae, it is impossible to maintain the life cycle in the laboratory, and clinical samples must be used for research. Even for those filarial nematodes that infect non-human vertebrates, the life cycle is difficult to maintain in the laboratory since it requires both the vertebrate and invertebrate hosts. For that reason, a zoonotic isolate of *B. malayi* [3] is the only filarial parasite of medical importance that is routinely used for research purposes. Therefore, the availability of high quality genome sequences from other filarial nematodes provides an alternate starting point to explore the biology of these organisms.

There have been numerous challenges in sequencing filarial nematode genomes. Clinical specimens have to be obtained from remote areas where the infection is endemic. Such samples often contain a limited quantity of nucleic acids of varying quality, complicating library construction. These samples also contain a population of genetically heterogeneous genomes with polymorphisms that complicate assembly. Furthermore, the genomes are large and have been difficult to assemble, owing to numerous repeats, low complexity sequences, and a nucleotide content of 30% GC [1, 2]. Previous difficulties in assembling *B. malayi* were attributed [1] to the repeat content of the genome, which was estimated at ~15% [1, 4]. Likewise, ~9% of the *L. loa* genome was estimated to be in repeats [2]. An analysis of the *B. malayi* genome suggested that the sequences in gaps had a higher AT content [1]. In *B. malayi*, a large amount of sequence integrated into the nematode nuclear genome from its obligate mutualist *Wolbachia* endosymbiont relatively recently such that it has not diverged significantly [5, 6], which further complicates assembly, resulting in numerous collapsed repeats and corresponding gaps in the assembly [5]. Unlike other human filarial parasites, *L. loa* does not have *Wolbachia* endosymbionts [7] and has very minimal amounts of *Wolbachia* DNA integrated in its nuclear chromosome [2]. This alleviates one of the issues described above that has complicated filarial genome assemblies.

*L. loa* is the causative agent of loiasis (“African eye worm”) and is transmitted by *Chrysops* spp. (deerflies). Historically, *L. loa* has not been the best-studied of the medically relevant filarial nematodes [2], partly because infected individuals in endemic areas are typically clinically asymptomatic [8]. However, in 1995, mass drug administration (MDA) campaigns began that were aimed at interrupting transmission of lymphatic filariasis and onchocerciasis. MDA campaigns that used ivermectin to target onchocerciasis in areas where the population was co-infected with *L. loa* had an increased incidence of severe adverse events including encephalopathy and death [9]. Therefore, the *L. loa* genome has clinical importance as well as providing a backdrop for developing better molecular diagnostics to ensure success of the global programs to eliminate lymphatic filariasis and onchocerciasis.

The introduction of the Pacific Biosciences platform in 2011 provided an opportunity to leverage its significantly longer reads to improve the *L. loa* genome sequence and assembly and to generate a more complete filarial nematode genome. The prior genome was sequenced from material collected from a patient from Cameroon. Here, we undertook the sequencing of a second *L. loa* clinical specimen from an individual from the Central African Republic. Both of these countries are within the 10 countries that are at the center of *L. loa* infection in Africa [10], but the genomes should be polymorphic with respect to one another. Using this sample, we describe 96.4 Mbp in 2,250 contigs, which represents ~9% more of the genome in ~85% fewer contigs from ~80% less starting material. The result is the most complete filarial nematode assembly published thus far at a fraction of the cost of previous efforts.

## Results

### Sequencing results

To improve the *L. loa* genome, whole genome sequencing was undertaken using the Illumina MiSeq and Pacific Biosciences (PacBio) RS II platforms. The previously published *L. loa* genome was sequenced with Roche 454 FLX reads resulting in 91.4 Mbp in 5,773 scaffolds divided between 3.8 Mbp of gaps and 87.5 Mbp of consensus sequence in 14,332 contigs (Tables 1 and 2) [2]. We sought to improve this using the latest sequencing strategies. We were able to obtain ~10 μg of high quality, high molecular weight DNA from a clinical specimen containing microfilariae of *L. loa*. The Illumina MiSeq library that was constructed had an estimated insert size of 646 bp and was sequenced on the MiSeq to generate 5.2 Mbp from >8.7 million 301-bp paired end reads. The PacBio library that was constructed had an estimated mean insert size of 8.4 kbp and was sequenced on 25 SMRT cells resulting in 78.85× raw sequencing coverage with 7.6 Gbp of sequence from >1.8 million reads with an estimated maximum read length of 25 kbp, a mean read length of ~4.1 kbp, and a median read length of ~3.6 kbp.

**Table 1:** **Assembly statistics for scaffolds**

Assembler	Sequencing data	No. scaffolds	Total length	No. scaffolds (≥1 kbp)	Total length (> = 1 kbp)	Largest scaffold	GC (%)	N50
Newbler	454	5,774	91.4	NR	NR	NR	31.0	172,000
CA	Illumina MiSeq	2,902	86,081,730	2,891	86,076,258	447,471	30.7	64,834
MaSurCA	Illumina MiSeq	27,116	96,961,118	16,611	90,177,928	129,528	30.8	10,831
CLCBio	Illumina MiSeq	72,775	104,010,594	12,719	78,215,812	115,234	29.3	7,816
CA	Illumina-corrected PacBio reads	4,436	94,016,717	4,436	94,016,717	901,957	31.0	61,260
CA	Illumina- and 454-corrected PacBio reads	4,222	100,293,095	4,222	100,293,095	905,793	30.8	89,633
HGAP2	PacBio reads	2,601	102,405,157	2,601	102,405,157	1,570,895	30.7	163,655
HGAP2 with trimming	PacBio reads	2,250	96,410,008	2,183	96,369,305	1,570,872	30.8	180,288

This table provides the statistics about the scaffolds generated by various combinations of assemblers and data sets. The statistics reported include the number of scaffolds, total length of sequence, number of scaffolds ≥ 1 kbp, the total length of scaffolds ≥ 1 kbp, the size of the largest scaffold, the percent GC, and the N50. The previously published values for the 454-based assembly (Genbank: ADBU02) is included for comparison; NR denotes that the value was not reported.

**Table 2:** **Assembly statistics for contigs**

Assembler	Sequencing data	No. contigs	Total length	No. contigs (≥1 kbp)	Total length (≥1 kbp)	Largest contig	GC (%)	N50
CA	Illumina MiSeq	3,785	86,045,090	3,670	85,991,832	362,075	30.7	49,810
MaSurCA	Illumina MiSeq	27,180	96,959,781	16,645	90,159,179	129,528	30.8	10,719
CLCBio	Illumina MiSeq	72,775	104,010,594	12,719	78,215,812	115,234	29.3	7,816
CA	Illumina-corrected PacBio reads	4,436	94,016,717	4,436	94,016,717	901,957	31.0	61,260
CA	Illumina- and 454-corrected PacBio reads	4,222	100,293,095	4,222	100,293,095	905,793	30.8	89,633
HGAP2	PacBio reads	2,601	102,405,157	2,601	102,405,157	1,570,895	30.7	163,655
HGAP2 with trimming	PacBio reads	2,250	96,410,008	2,183	96,369,305	1,570,872	30.8	180,288

This table provides the statistics about the contigs generated by various combinations of assemblers and data sets. The statistics reported include the number of contigs, total length of sequence, number of contigs ≥ 1 kbp, the total length of contigs ≥ 1 kbp, the size of the largest contig, the percent GC, and the N50.

### Assembly results from PacBio and Illumina data

**Figure 1:**
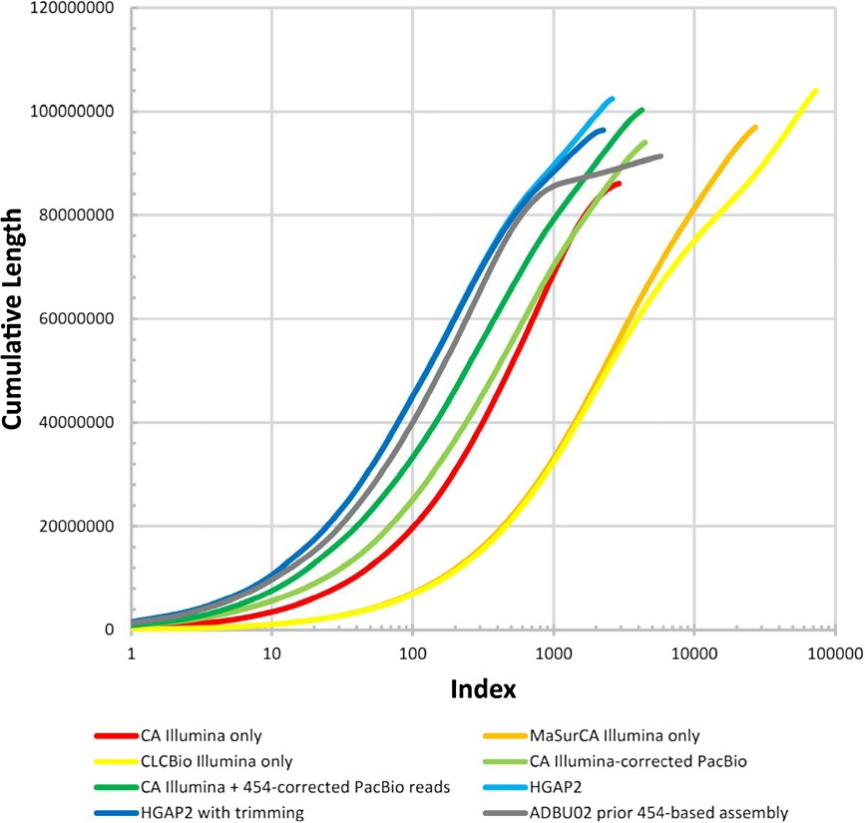
**Cumulative length of contigs.** The cumulative length of the assemblies are plotted for all contigs. Contigs are indexed (*x*) from longest to shortest. The cumulative length of the first *x* contigs are plotted as a function of *x* for the assembly using a log-transformed x-axis.

The sequencing reads were assembled with six different strategies: (1) Celera assembler (CA) [11] with only Illumina MiSeq data; (2) MaSuRCA assembler [12] with only Illumina MiSeq data; (3) CLC assembler (CLC bio, Cambridge, MA, USA) with only Illumina MiSeq data; (4) CA with PacBio reads corrected with Illumina MiSeq data [13]; (5) CA with PacBio reads corrected with Illumina MiSeq data generated here and the Roche 454 data previously published [2], and (6) HGAP2 with only the PacBio reads [14]. QUAST [15] was used to compare the assemblies by aligning them to the previously published *L. loa* genome (Genbank: ADBU02) [2]. Based on total length, number of scaffolds/contigs, largest scaffold/contig length, and scaffold/contig N50, HGAP2 outperformed the other approaches for both scaffolds (Table 1) and contigs (Table 2). HGAP2 only generates contigs, so the values for contigs and scaffolds are identical. HGAP2’s superior performance relative to the other methods tested was further supported by plots of the cumulative length of the assembly as a function of contigs sequenced (Figure 1).

Contigs and scaffolds were further examined following positive filtering to remove contigs that did not match the previously published *L. loa* genome. Such filtering would remove contaminants as well as sequence from the human host. Positive filtering removed a small percentage of contigs in all cases, but the overall assembly statistics remained similar. The HGAP2 assembly of only PacBio reads still outperformed all other methods examined with the largest genome size, largest N50, and the fewest number of contigs. Manual curation of the contigs removed by positive filtering from the HGAP2 assembly revealed sequences consistent with being in the *L. loa* genome, meaning they did not currently have any other matches in NT and often contained low complexity repeats. Therefore, the HGAP2 assembly without positive filtering was selected for further examination and validation.

### Validation and trimming with quiver

**Figure 2:**
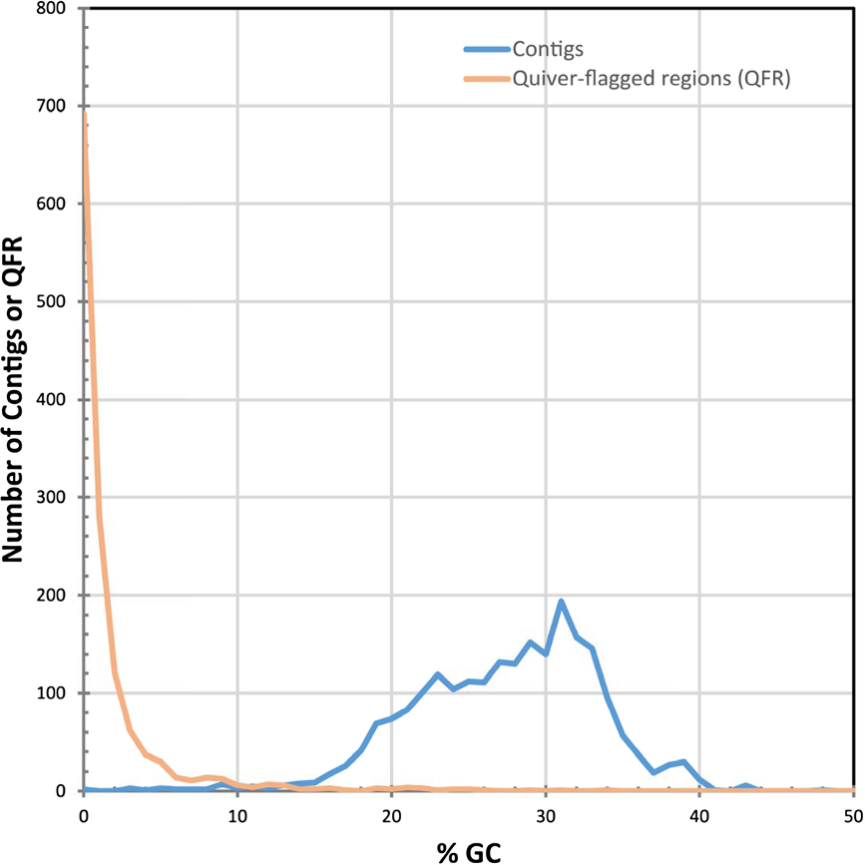
**GC coverage of high and low confidence areas.** Histograms of the %GC for all contigs and all internal regions flagged by Quiver are presented. Many of the internal regions that are poorly supported are AT-rich relative to the entire set of contigs.

PacBio contigs can end with erroneous sequence that results from the joining of a single chimeric fragment, which is frequently and easily detected in bacterial genomes that are sequenced to completion and need to be circularized (Dunning Hotopp, personal observation). Such regions should be detected with PacBio’s Quiver algorithm, which finds the maximum likelihood consensus *de novo* using the sequence as well as numerous covariates provided by the basecaller. To examine the extent to which poorly supported sequences at contig ends increases the predicted genome size, we examined the contig containing the circular *L. loa* mitochondria genome, which had a 6 kbp overlap before circularization. Beyond this overlap, there was ~450 bp and ~1200 bp of erroneous sequence on the left and right flanks, respectively. This was also identified with Quiver, which flagged 208 bp and 1416 bp of sequence as being poorly supported on the left and right flanks, respectively. This indicates that Quiver can identify and estimate erroneous extra sequences that may be artificially inflating the genome size. With the HGAP2 assembly, 7.89 Mbp of data were flagged by Quiver as having lower confidence. Trimming off Quiver-flagged regions from the ends of contigs and removing resulting contigs <200 bp in the 102.4 Mbp HGAP2 assembly yielded a 96.4 Mbp assembly. In this way, the actual genome size may be smaller than the HGAP2 assembly size because there are large regions with lower confidence at contig ends, which likely scales with the number of contigs in the assembly. All subsequent analyses were performed on this Quiver-trimmed HGAP2 assembly, which was deemed to be the final assembly and the most accurate assessment of the genome size. Providing further validation of the quality of this assembly, CEGMA3 analysis [16] identified 237 (95.6%) complete genes of the 248 core eukaryotic genes in this assembly, while an additional 6 partial genes were identified (98%). This is slightly more than the 236 complete and 5 partial core eukaryotic genes found in the previous *L. loa* assembly.The remaining data flagged by Quiver that was internal to the contigs were frequently low complexity, AT-rich trimeric and tetrameric repeat arrays. Consistent with this, Quiver-flagged internal regions had a lower GC-content than the rest of the genome (Figure 2).

### Differences in assembly content

**Figure 3:**
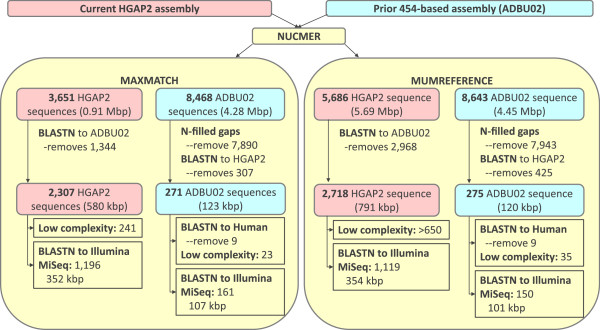
**Assembly differences.** The differences between the HGAP2 assembly presented here and the prior 454-based assembly are examined using NUCMER and BLASTN. The larger number of sequences uniquely in the HGAP2 assembly and the larger number of low complexity sequences suggests that the PacBio sequence data resolves more of the genome by spanning low complexity repeats. Some regions were identified in the 454-based assembly that were in the Illumina MiSeq assembly, suggesting that the sequences are missing from the HGAP2 assembly.

In order to assess the genome content differences between the prior 454-based assembly and the current Quiver-trimmed HGAP2 assembly, we identified the gaps in both assemblies using NUCMER separately with the MUMREFERENCE and the MAXMATCH options followed by a subsequent BLAST search against the same database (Figure 3). The subsequent BLAST search was needed because NUCMER identified some sequences up to 18 kbp in length as missing in the reference that were actually present in the reference. The results using MAXMATCH and MUMREFERENCE with a subsequent BLASTN search were congruent (Figure 3).

For the current HGAP2-assembled sequence, 2,307-2,718 unique sequences spanning 580–791 kbp were identified as not present in the prior 454-based assembly using NUCMER with MAXMATCH or MUMREFERENCE followed by a subsequent BLASTN against the prior 454-based assembly (e-value < 0.000001). Of these, hundreds were low complexity sequences that were not likely resolved with the 454-based sequencing.

As mentioned previously, the 454-based assembly had a significant number of N-filled gaps. Not surprisingly then, of the 8,468-8,643 “sequences” found only in the 454 assembly but not the PacBio assembly that span 4.28-4.45 Mbp, the vast majority contain Ns. Of the 578–700 sequences without Ns, 307–425 had a match with BLASTN (e-value < 0.000001) in the HGAP2 assembly. Of those remaining 271–275 sequences spanning 120–123 kbp, 9 have matches in NT to the GRCh37 release of the human genome reference (e-value < 0.000001), 23–35 are low complexity, and the remainder have no significant homology to anything except *L. loa* and other nematodes in NT.

These results suggest that both the prior assembly of 454 data and the current assembly of PacBio data had unique regions not covered by the other assembly. These could be due either to gaps in the sequencing and assembly, biological variation, or both. Of the 271–275 sequences in the 454-based assembly but not the HGAP2 assembly, 150–161 spanning 101–107 kbp had a match to the Illumina MiSeq assembly (e-value e-15). This suggests those sequences are merely missing from the HGAP2 assembly, since the DNA source was the same for the Illumina MiSeq and HGAP2 assemblies. These sequences were not identified in the HGAP2 assembly prior to Quiver-based trimming indicating that the sequences were not erroneously removed during trimming. The larger number of sequences uniquely in the HGAP2 assembly as well as the larger number of low complexity sequences suggests that, as expected, the PacBio sequence resolved more of the genome by spanning low complexity repeats.

Nine contigs with significant homology to human sequences could be identified in the prior 454-based assembly and have now been removed from the genome deposited at NCBI (Desjardins, personal communication). No sequences with homology to human sequences were identified in the PacBio assembly presented here. More specifically, no sequence in the Quiver-trimmed HGAP2 assembly was identified with >95% identity across 100 bp of sequence using BLASTN and the GRCh38 release of the human genome.

### Validation with Illumina data

**Figure 4:**
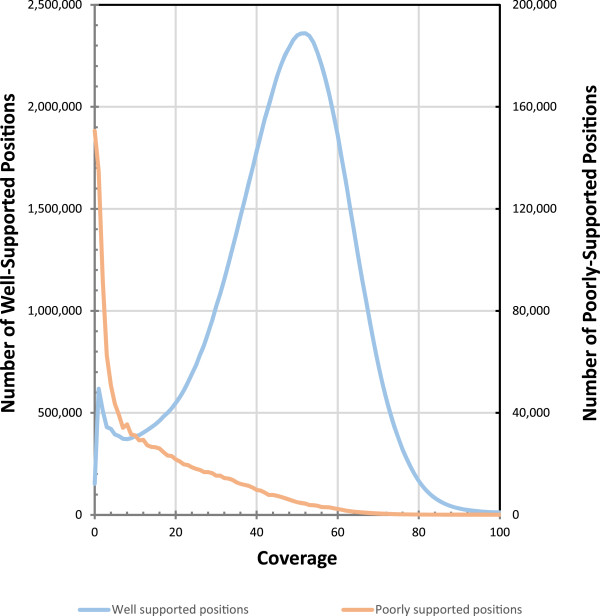
**Sequencing coverage distribution of high and low coverage areas.** Histograms of the coverage at each position following mapping of the Illumina MiSeq reads to the Quiver-trimmed HGAP2 assembly are plotted for those positions that Quiver identifies as well-supported and those that are poorly-supported. Many of the remaining poorly supported internal positions have low coverage.

In order to further validate the Quiver-trimmed HGAP2 assembly, we mapped all of the Illumina MiSeq reads to it. The Illumina sequence data was derived from the same DNA sample as the PacBio data, so we expect similarities. Approximately 307 kbp of the assembly had 0× coverage in Illumina reads (Figure 4), which was evenly split between positions in the internal regions that were flagged by Quiver (154,504 positions) and positions in regions that were not flagged by Quiver (152,446 positions). If positions with ≤2× coverage are examined, three times the number of positions are identified in regions not flagged by Quiver (1,281,258 positions) than those flagged by Quiver (395,098 bp). However, considering that <2% of positions were flagged by Quiver relative to those not flagged by Quiver in the assembly, this demonstrates a congruence between internal regions flagged by Quiver and coverage as assessed by the Illumina mapping.

**Figure 5:**
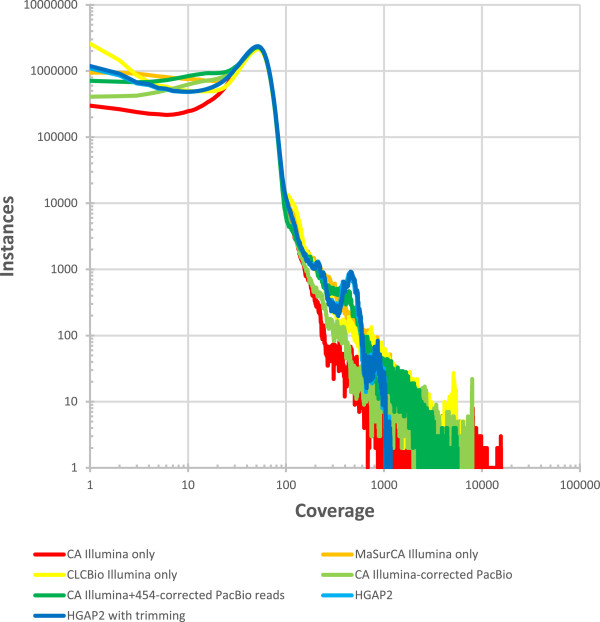
**Sequencing coverage distribution.** A histogram of the coverage at each position following mapping of the Illumina MiSeq reads to all assemblies are plotted with log-transformed axes. Regions with abnormally little coverage exist, as well as those with excess coverage, for all assemblies. The peak in coverage for the Illumina data was at 51× coverage. In addition to this major peak, two additional peaks were identified at 200× and 450× in the HGAP2 assembly, reflecting collapsed repeats in the assembly including the mitochondrial genome, a *L. loa* interspersed repeat, and the rRNA.

The peak in coverage for the Illumina data was at 51× coverage (Figures 4 and 5). In addition to this major peak, two additional peaks were identified at 200× and 450× (Figure 5), which could reflect collapsed repeats in the assembly. An examination of the regions with >350× coverage revealed that the contig with the most positions in this coverage range contained the complete mitochondrial genome. This result likely reflects the numerous mitochondrial genomes sequenced for every one nuclear genome sequenced. The next two contigs in rank abundance of positions with >350× coverage contained a *L. loa* interspersed repeat and the rRNA. A similar examination of the three contigs with the most positions at 180-250× coverage revealed contigs also containing *L. loa* repeats. There are two possible explanations for this result. This may reflect the continued presence of collapsed repeats in the assembly despite the long sequence reads. Alternatively, the result may stem from some regions being better corrected than other regions. For identical or nearly identical repeats, Illumina reads will map better to a corrected repeat than an uncorrected repeat. In this way, the true coverage may be inadequately divided between some high coverage areas and low coverage areas.The NUCMER searches described above demonstrate that the PacBio sequences can better resolve repeats. Therefore, as expected, the HGAP2 assembly had the lowest maximum coverage value (~1,100×, or 22× the median coverage) of all assemblies while the assemblies that relied on only MiSeq data have the highest maximum coverage (~15,000×, or 300× the median coverage) (Figure 5). The HGAP2 assembly is also the only assembly to have noticeable peaks in the coverage distribution reflecting collapsed repeats including the mitochondria and rRNA as discussed above. It is less clear why these coverage peaks are not visible in the coverage distributions for the other assemblies. It may be that the repeats are not as well resolved. In particular, if they are unequally fragmented in the assembly, the Illumina sequence reads will be unequally distributed when mapping with BWA.

Several metrics were also calculated on the alignments using PICARD including the number of discordant read pairs (Table 3). The number of discordant read pairs was similar between all the assemblies and ranged between 0.02-0.2% of the total reads. The similarity of the results across Illumina-only and PacBio-only assemblies may indicate that the discordance measured reflects sequencing errors as opposed to assembly errors.

**Table 3:** **Mapping metrics for assemblies**

Assembler	Sequencing data	FR reads	RF reads	Tandem	Median insert Size ± Abs. Dev.	Mean insert Size ± St. Dev.
CA	Illumina MiSeq	7,163,609	690	739	470 ± 45	480 ± 72
MaSurCA	Illumina MiSeq	7,315,963	340	137	469 ± 44	478 ± 71
CLCBio	Illumina MiSeq	6,917,178	103	29	466 ± 44	475 ± 72
CA	Illumina-corrected PacBio reads	7,292,995	694	298	470 ± 44	479 ± 71
CA	Illumina- and 454-corrected PacBio reads	7,459,123	600	288	470 ± 44	480 ± 71
HGAP2	PacBio reads	7,544,255	696	826	471 ± 45	480 ± 71
HGAP2 with trimming	PacBio reads	7,564,549	697	827	471 ± 45	480 ± 71

This table provides the statistics about the mapping of all of the Illumina read pairs against all of the contigs generated by various combinations of assemblers and data sets as assessed by PICARD. The statistics reported include the number of FR reads, the number of RF reads, the number of tandem reads, the median insert ± on absolute deviation, and the mean insert size ± one absolute deviation.

### Heterogeneity

**Figure 6:**
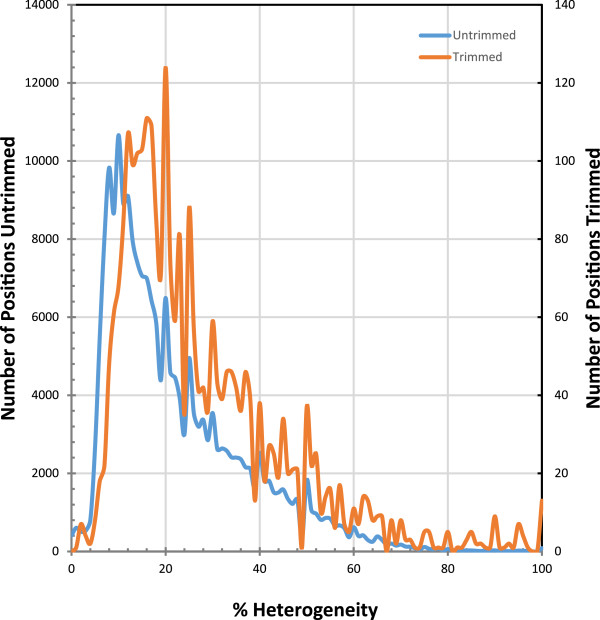
**Percentage of heterogeneity for SNPs in high and low confidence regions.** Histograms are presented for the percent heterogeneity of SNPs. Since the population is not intentionally inbred, mixed levels of polymorphism are expected at a variety of positions. A SNP was defined as a position with >20× coverage that had polymorphisms supported by >3 reads. Regions that Quiver identified as being supported and those flagged as having low support were examined separately and were found to have similar profiles. While the HGAP2 consensus typically contained the base supported by the majority of reads, there are regions where 100% of the reads mapping to the assembly support an alternative call.

**Figure 7:**
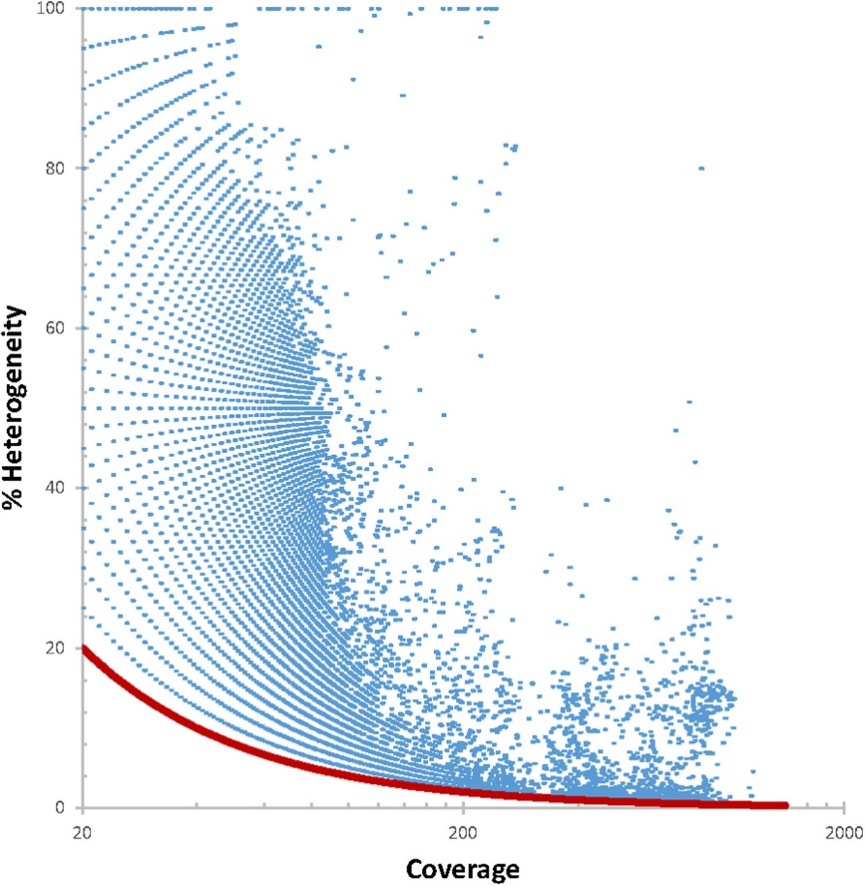
**Relationship between coverage and percent heterogeneity.** A SNP was defined as a position with >20× coverage that had polymorphisms supported by >3 reads. For each position with a SNP, the percent heterogeneity and coverage are plotted. The red line shows the minimum bounds of the data that is defined by requiring >20× coverage and polymorphisms in three reads. As expected, some of the 100% heterogenous positions have higher coverage than average suggesting that they may be collapsed repeats. This plot does not enable any conclusions about the frequency at which a value occurs since data points with the same values are only plotted once.

**Figure 8:**
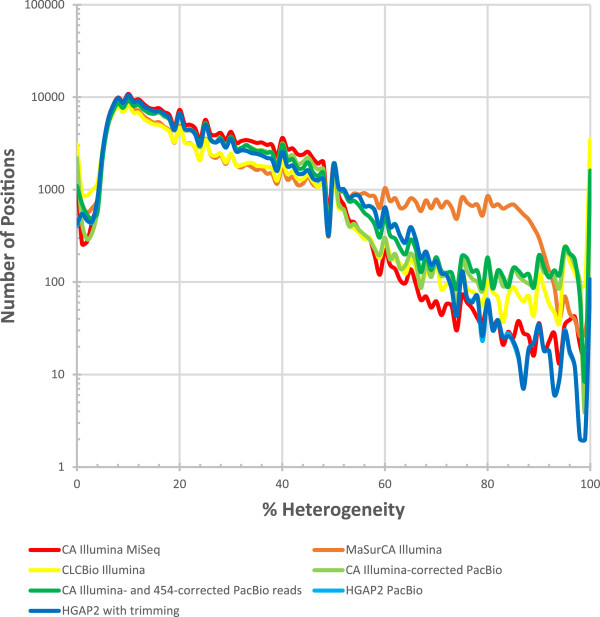
**Percentage of heterogeneity for SNPs in multiple assemblies.** Histograms are presented for the percent heterogeneity of SNPs. Since the population is not intentionally inbred, mixed levels of polymorphism are expected at a variety of positions. A SNP was defined as a position with >20× coverage that had polymorphisms supported by >3 reads. While one might anticipate minimal or no positions at >50% heterogeneity, numerous such instances are observed for all of the assemblers tested with all datasets, including the assemblies based solely on the same Illumina data that was used for the mappings. HGAP2 performed similar to, or better than, the other assemblers examined.

Since there is no animal host for *L. loa* and the sample was taken directly from a patient, there was no intentional inbreeding of the nematode to generate a homozygous line. Thus, we expect to identify polymorphic positions within this DNA sample. To examine the single nucleotide polymorphism (SNP) heterogeneity, a SNP was defined as a position with >20× Illumina MiSeq coverage that had SNPs supported by >3 reads that reflected substitution mutations as identified by SAMTOOLS and no indication of the presence of an insertion or deletion (indel). There are 83,737,752 such positions that have >20× coverage and no indel. Of those, 206,137 positions, or 0.2% of the positions, have >3 reads supporting the same alternate polymorphism. The HGAP2 consensus call was most frequently consistent with the majority of Illumina reads. However, there were regions where 100% of the Illumina reads mapping to the HGAP2 assembly supported an alternative call (Figure 6) despite both libraries being constructed from the same DNA. The regions that were identified as poorly supported using Quiver had a similar distribution of polymorphisms as the regions that were identified as supported (Figure 6) suggesting that support, as assessed by Quiver, was not the sole source of this error. One ~6 kbp contig contained the most such positions, with 25 positions across an ~1.1 kbp stretch where 100% of the mapped Illumina reads disagreed with the HGAP2 consensus call. This 1.1 kbp region can be identified in four separate contigs in the previously published genome [2] suggesting that repeats are one source of this problem. Further supporting that repeats may be one source of this error, some of the 100% heterogenous positions have higher coverage than average suggesting that they may be collapsed repeats (Figure 7).Such SNP heterogeneity as a function of coverage is very similar across all of the assemblies, with the exception of the MaSurCA assembly of only Illumina reads, which had a higher number of positions with 50-85% heterogeneity (Figure 8). Of note, we did not optimize any of the assemblies, so it is possible that MaSurCA could perform better with optimization. With regard to positions that were 100% discordant between the consensus base and the Illumina reads, MaSurCA had the fewest with only 86 such positions and CLCBio had the most with 3,501, followed by CA assembly of Illumina- and 454-corrected PacBio reads with 1,612 and CA assembly of Illumina-corrected PacBio reads with 1,455. Both trimmed and untrimmed HGAP2 assemblies had 107 such positions. Therefore, all of the assemblies had such positions and HGAP2 did not perform significantly worse than the other assemblers. The Illumina-reads mapped against the Illumina-only assembly with CA had 98 such positions, which is similar to the 107 such positions found in the HGAP2 assembly.

## Discussion

### PacBio sequencing of a metazoan population

Genome sequencing platforms are improving at a rapid pace, providing access to more and better genomes at a reduced cost. An era of short reads with small insert sizes enabled re-sequencing efforts for model organisms and humans with large genomes. However, *de novo* sequencing of large genomes from non-model organisms languished. Capillary-based sequencing remained cost prohibitive, but until recently, newer technologies did not offer the combination of read length and insert size needed to *de novo* assemble large genomes. However, single-molecule, real-time sequencing using the PacBio RS II generates long sequence reads and provides an opportunity to generate new, high-quality genome sequences from non-model organisms with large genomes, as well as improve the assemblies of those that have been sequenced previously.

Many of the improvements realized by PacBio have already been applied to bacterial genomes. The platform was used for rapid sequencing of disease outbreak strains including the Haitian cholera outbreak in 2010 [17] and the German *E. coli* outbreak in 2011 [18]. Subsequently, numerous bacterial genomes have been sequenced, many of which are closed solely with PacBio data [19]. More recently, PacBio sequencing has been applied to resolving segmental duplications in primate genomes [20]. However, to our knowledge, successful *de novo* sequencing of large non-model metazoan genomes using the PacBio platform has not yet been published.

Here, we demonstrate that the sole use of PacBio reads yields a larger genome assembly with fewer gaps than similar genomes generated through capillary sequencing or 454 sequencing. For example, 9× coverage using capillary based sequencing of 1.6-3.0 kbp and 10–12 kbp libraries as well as fosmids and BACs for *B. malayi* yielded 77.5 Mbp in 8,180 scaffolds with an N50 of 94 kbp and 17.5 Mbp in degenerate contigs [1, 4]. The sequencing for that project alone likely cost >$1 million. For *L. loa,* Roche 454 FLX sequencing of shotgun libraries and a 3-kbp jumping library to a combined 20× coverage yielded a 91.7-Mbp assembly with 5,774 scaffolds with an N50 of 172 kbp [2]. Given that 18 Roche 454 sequencing runs were deposited at the Sequence Read Archive, the sequencing alone would have cost >$50,000, and likely cost at least twice that. In both cases, the number of gaps were significant and the amount of sequence data in contigs was significantly less than the aggregate size of the scaffolds, with 70.7 Mbp and 87.5 Mbp of sequence in 26,589 and 14,332 contigs for *B. malayi* and *L. loa,* respectively. Assembly of PacBio data yields only contigs, since reads are not paired, but those contigs are larger than the scaffolds reported previously, suggesting many of the previous gaps are now sequenced. The Quiver-trimmed HGAP2 assembly generated here is 96.4 Mbp in 2,250 contigs and the sequencing cost <$12,000. The raw PacBio sequencing coverage was 78.85× and the PacBio-corrected bases in the assembly yielded 16.4× coverage. The resulting assembly contains 8.9 Mbp more sequence (~9% of the total size) in ~85% fewer contigs at a fraction of the cost, and is the most completely assembled filarial nematode genome published thus far. Furthermore, the most recent assembly required 80% less starting material (<10 μg of genomic DNA) than the Roche 454-based sequencing project, which reported using ~50 μg [2], which is an important consideration for clinical samples.

### Need for further improvements in polishing algorithms

While the result is a more complete genome, it still contains some misassemblies and errors. Misassemblies were identified with Quiver that could reflect real rearrangements as well as artifacts resulting from ligation of genomic DNA fragments during library construction. The library construction protocol relies on a blunt end ligation to add adaptors to genomic DNA fragments. Ligation occurs in an excess of adaptors, which will favor ligation of adaptor to fragment ends. However, the inappropriate joining of two fragments can also occur. We expect each such pair of ligated fragments to be unique. As such, more sequencing coverage would ultimately overwhelm such chimeric reads resulting in their effective removal from assemblies except at contig ends. Therefore, more sequence coverage would be expected to yield a more complete genome. However, we used the entire DNA sample available, so we could not obtain additional sequence coverage to improve this assembly.

Additionally, we identified a number of Quiver consensus calls poorly supported by the aligned Illumina MiSeq data. We explored using Illumina reads to correct the PacBio consensus using the consensus calling available in SAMTOOLS. However, the repetitive nature of the genome precluded such an undertaking with many regions lacking uniquely mapping reads. Furthermore, it is unclear whether the Illumina data or the PacBio consensus would be more accurate given that we observed the poorly supported consensus calls in all of the assemblies generated.

To investigate this further, we excised the 100 bp upstream and downstream of the 107 positions that were 100% heterogeneous in the HGAP2 assembly and compared them to the prior 454-based assembly. Most of these sequences were present at least twice in the genome with both polymorphisms present between the paralogs. Therefore, the most likely source of this error in all the assemblies is the inability of the assembler to adequately resolve paralogs, which is also a problem in the BWA-based mappings.

In HGAP2, we suspect that the error correction step may be the source of this error. Under this scenario, some SNPs may be erroneously corrected to match a paralog while others are not. The result is a hybrid sequence like those we observe in the HGAP2 assembly. In that case, the Illumina sequences for the two paralogs will quite possibly be unequally distributed between the two paralogs. Unfortunately, HGAP2 does not save the alignments used for the error correction step to investigate this further. However, one can imagine that a Quiver-like polishing algorithm that uses multiple sequence types and their quality values that is able to take into account each sequence type’s inherent biases may yield the most error-free assembly, particularly if the algorithm better focuses on resolving paralogs, possibly through contextual based error correction as in chromosome phasing algorithms. This may be particularly useful for clinical samples where DNA quantities are limited, homozygous lines are not available, and further sequencing may not be possible.

## Conclusions

Here, we demonstrate the ease of generating an economical genome assembly using high-quality, long-read PacBio data from genetically heterogeneous filarial nematode DNA from a clinical sample. A comparison of the sequence data types and assembly reveals that HGAP2 assembly of PacBio data alone yields the most complete genome of the assembly methods tested. Applying this strategy to further samples, as well as to other medically important metazoans, is likely to be fruitful. The differences between this assembly of *L. loa* and the one that was published previously, likely reflect differences in the *L. loa* populations sequenced, at least in part. Therefore, we look forward to the analysis of this more complete genome sequence in order to improve our understanding of loiasis and the genomic diversity of *L. loa*. Future directions include examining the two genomes and the encoded proteins more systematically and understanding population variation and selection. In addition, these two robust assemblies could be assembled to create a more complete non-redundant consensus reference *L. loa* genome, using the methods established for the generating the non-redundant consensus human genome.

## Methods

### Isolation of *L. loa*microfilariae

During a therapeutic apheresis, 5 × 10^5^ microfilariae were purified from a patient with loiasis infected in the Central African Republic seen at the NIH under protocol 88-I-83 (NCT00001230) approved by the NIAID Institutional Review Board. Written informed consent was obtained from the patient. All protocols and consenting were in compliance with the Helsinki Declaration for the protection of human subjects. Purification of the microfilariae occurred following centrifugation over a ficoll-diatrizoate density gradient followed by filtration through a 3 μM filter. The filters were rinsed in RPMI supplemented with antibiotics and the microfilariae that were able to swim off were collected, rinsed, centrifuged, and stored in liquid nitrogen until used.

### Preparation of genomic DNA

The cryopreserved purified microfilariae were thawed, and then rinsed twice in PBS and once in 1× DNase I Buffer (Roche) before being resuspended in 200 μL of DNase I Buffer containing 100 U of DNase I for 1 h at 37°C to remove any free human DNA. The sample was washed twice in PBS by centrifugation at 2000 rpm for 10 min. The microfilariae were resuspended in 180 μL of ATL buffer (Qiagen) and vortexed for 10 s. Proteinase K (20 μL) was added, and then the sample was vortexed for 30 s and incubated at 56°C for 1 h. The sample was processed using a DNeasy Kit (Qiagen) following the manufacturer’s protocol.

### Illumina library construction and sequencing

A genomic DNA library was constructed for sequencing on the Illumina platform using a modified version of the KAPA Library preparation Kit (Kapa Biosystems, Woburn, MA, USA). DNA was fragmented with the Covaris E210. AMPure XT beads (Beckman Coulter Genomics, Danvers, MA, USA) were used to purify between enzymatic reactions and size select the library. The PCR amplification step was performed with primers containing a 6-mer index sequence. The library was sequenced on a multiplexed 301-bp paired-end run on an Illumina MiSeq, resulting in ~8.7 million passed filter read pairs.

### Pacific biosciences library construction and sequencing

Genomic DNA (~9.4 μg) was sheared using the Covaris gTube (Woburn, MA, USA). A PacBio sequencing library was constructed using the resulting ~5 μg of the sheared material and prepared for sequencing using the DNA template prep kit 2.0 (Pacific Biosciences, Menlo Park, CA, USA). Small library fragments were removed using the BluePippin (Sage Science, Beverly, MA, USA), resulting in an estimated mean insert length of 8.4 kbp. The library was loaded onto 25 v2 SMRT Cells and sequenced with polymerase P4 and sequencing chemistry C2 (Pacific Biosciences), resulting in a total of ~1.8 million sequence reads and 7.6 Gbp of passed-filter data.

### Assembly

The Illumina MiSeq data alone was assembled using CLC assembler v4.1.0, Celera Assembler v7.0, and MaSuRCA, a version of Celera Assembler that constructs merged super-reads prior to assembly. Using the latest PBcR (PacBio-Corrected Reads) correction algorithm, Illumina reads were aligned alone, or in combination with the previously published Roche 454 reads [2], to long PacBio reads and used to correct errors in the PacBio sequences. The resulting corrected PacBio reads were assembled with Celera Assembler v8.1. Finally, PacBio data alone was assembled using HGAP2, which uses shorter PacBio reads in a multiple alignment to correct errors in longer reads, which are then assembled. For the HGAP2 assembly, Quiver was used for consensus polishing. Specific parameters for each assembly are provided (Additional file 1). To generate the Quiver-trimmed HGAP2 assembly that was identified as the final assembly, Quiver-flagged regions at the ends of contigs were removed as well as resulting contigs <200 bp. We would have liked to compare HGAP2 to Celera Assembler for using shorter PacBio reads to correct errors in longer reads and to assemble the corrected-PacBio reads. To this end, we attempted a CA assembly with the PacBio reads at least eight times using different parameters as well as different hardware, but the assembler consistently failed at the overlapping step.

### Positive filtering

Following assembly, contigs were filtered using a MEGA-BLAST screen against the previously published *L. loa* genome [2] to select for contigs that align >95% over 100 bp in an effort to eliminate human contigs and other contaminants.

### Mapping Illumina data

Illumina reads were mapped against the Quiver-trimmed HGAP2 assembly using ALN/SAMPE in BWA [21] with default parameters. Duplicate reads were removed following sorting with Picard [22] using default parameters. Coverage values and SNPs were calculated and identified with MPILEUP in SAMTOOLS [23] using default parameters.

### Identifying differences between genomes

NUCMER [24] searches with both MAXMATCH and MUMREFERENCE were used to find differences between the prior 454-based assembly and the current PacBio-based assembly. After we identified numerous issues that resulted in misidentifying gaps, the “-c 120 -l 40” options were used to minimize clustering of unique anchors due to the high similarity of the HGAP2 assembly and the 454-based assembly. This requires a minimum cluster length of 120 (default = 65) and a minimum length of a maximal exact match of 40 (default = 20). The coordinates of matches were used to extract the sequences in nucmer-identified gaps. These sequences were searched against the reference assembly with BLASTN. Sequences without a BLAST match were extracted and searched against both NT and the CA Illumina assembly.

### Structural and functional annotation of *Loa loa*

Repeat masking was performed with RepeatMasker 4.0.5 [25, 26] using a library of characterized Nematoda repeats and a *de novo* one generated with RepeatModeler. *L. loa* transcriptome sequencing reads (>5 M spots, 872 M bases) [2] were downloaded from NCBI SRA (http://www.ncbi.nlm.nih.gov/sra/SRX130438), trimmed with Trimmomatic [27], and assembled with Trinity [28] using both *ab initio* and genome-guided approaches. An initial set of *ab initio* GeneMark-ES (GMES) [29] gene predictions was iteratively improved by incorporating these Trinity assemblies that were aligned to the genome using PASA [30] with BLAT and GMAP and with the TransDecoder option to estimate full length cDNAs. Complete GMES models with full length RNA-seq support were clustered with UCLUST [31] to remove 70% similar sequences and then searched against the NCBI non-redundant proteins using BLASTP [32]. Models matching non-*L. loa* proteins with e-values <1e-20 were retained for training with SNAP [33], Augustus [34], and GlimmerHMM [35]. After training, gene finders were run on genomic scaffolds. Augustus was run twice, once using RNA-seq intron hints mapped with Bowtie2/Tophat2 [36, 37] and once without hints, and non-overlapping models were combined. GeneID [38] was run using an existing parameter file. Protein alignments to *L. loa* genome were generated with AAT [39] against SwissProt and NCBI non-redundant database. EvidenceModeler (EVM) [40] was run using as inputs PASA alignments of Trinity RNA-seq assemblies, *ab initio* predictions, protein alignments, and repeat hints, all with manually assigned weights (RNA-seq > protein > *ab initio*). A second run of EVM was performed using only (a) equally weighted gene predictions from the five *ab initio* predictors and (b) heavily weighted gene predictions from the reference *L. loa* V3 annotation (http://www.broadinstitute.org/annotation/genome/filarial_worms) mapped to the genome with Exonerate [41] at 90% identity. Models from the second EVM run that were not overlapping models from the first were combined with the first to generate a full gene prediction set. Functional annotation was performed by querying predicted proteins with HMMER3 against a custom HMM collection that includes TIGRFams and PFam and searching against SwissProt with NCBI. These steps, in conjunction with custom-built databases of annotation assertions, provide assignments of gene product names, EC numbers, GO terms and gene symbols. Proteins significantly overlapping repeats were removed from the final annotation set. Lastly, predictions of non-coding RNAs generated with tRNAscan-SE [42] and RNAmmer [43] were added to the final set. Although CEGMA [16] was not used in structural annotation, CEGMA models were predicted in order to assess completeness of genome assembly.

### Availability of supporting data

The raw sequencing reads are deposited in the Sequence Read Archive SRA: SRP041627 (http://www.ncbi.nlm.nih.gov/sra/?term=SRP041627). The final Quiver-trimmed HGAP2 assembly is available in Genbank (Genbank: JPEI01000000).

## Electronic supplementary material

Additional file 1: **Recipes for genome assemblies.** A word document is provided outlining how each assembly was generated. (DOCX 16 KB)

## Abbreviations

CA: Celera assembler
Indel: Insertion or deletion
MDA: Mass drug administration
PacBio: Pacific Biosciences
SNP: Single nucleotide polymorphism.
