# Supplementary material for: Single molecule sequencing and genome assembly of a clinical specimen of Loa loa, the causative agent of loiasis
Source: BMC Genomics. 2014 Sep 12;15(1):788. doi: 10.1186/1471-2164-15-788 (PMC4175631; doi:10.1186/1471-2164-15-788)
Supplement: Supplementary file 1 — Additional file 1: Recipes for genome assemblies. A word document is provided outlining how each assembly was generated. (DOCX 16 KB) [file 12864_2014_6475_MOESM1_ESM.docx]

***Recipes for genome assemblies***

This section lists the assembler version and commands and parameters that we used for each assembler.

To run **Celera assembler (CA)** on Illumina reads only:

Version: 7.0

Command and parameters:

fastqToCA -insertsize 525 33 –libname reads -mates reads1.fastq,reads2.fastq > all_reads.frg

runCA –d . –p ca_illumina –s CA.spec utgGenomeSize=87000000 all_reads.frg>&runCA.log

with CA.spec specifying

unitigger = bogart

utgErrorRate = 0.025

utgBubblePopping = 1

bogBadMateDepth=110

merylMemory = 128000

ovlStoreMemory = 8192

ovlHashBits=24

ovlHashBlockLength=180000000

To run **MaSuRCA**, on Illumina reads only:

Version: 2.0

Commands and paramters:

runSRCA.pl config

./assemble

with config file specifiying:

PATHS

JELLYFISH_PATH=/full/path/to/ MaSuRCA-2.0/bin

SR_PATH=/full/path/to/ MaSuRCA-2.0/bin

CA_PATH=/full/path/to/ MaSuRCA-2.0/CA/Linux-amd64/bin

END

DATA

PE= pe 525 53 reads1.fastq.gz reads2.fastq.gz

END

PARAMETERS

USE_LINKING_MATES=1

CA_PARAMETERS = ovlMerSize=30 cgwErrorRate=0.25 ovlMemory=4GB

KMER_COUNT_THRESHOLD = 1

GRAPH_KMER_SIZE=auto

NUM_THREADS=16

JF_SIZE= 3000000000

DO_HOMOPOLYMER_TRIM=0

END

To run CLC-assembler on Illumina reads only:

Version: 4.1.0

Command and parameters:

clc_assembler --cpus 16 -o scaffolds.fasta -p fb ss 525 53 -q -i reads1.fastq.gz reads2.fastq.gz

To run **PBcR-Celera assembler (CA)** on PacBio reads + Illumina reads:

Version: Celera assembler 8.1

PBcR command and parameters:

pacBioToCA -length 100 -partitions 200 -l lib-name -t 12 -s pacbio.spec genomeSize=87000000 -fastq pacbio.fastq all_reads.frg

with pacbio.spec specifying:

maxCoverage=50

maxGap = 1500

blasr=-noRefineAlign -advanceHalf -noSplitSubreads -minMatch 10 -minPctIdentity 70 -bestn 24 -nCandidates 24

utgErrorRate = 0.25

utgErrorLimit = 6.5

cnsErrorRate = 0.25

cgwErrorRate = 0.25

ovlErrorRate = 0.25

unitigger = bog

ovlHashBits = 24

ovlHashBlockLength = 1000000000

ovlRefBlockLength = 1000000000

ovlRefBlockSize = 0

CA command and parameters:

runCA -p pbcr_illumina -d CA_all ovlMinLen=1000 kickOutNonOvlContigs=1 cgwDemoteRBP=0 cgwMergeMissingThreshold=0.5 -s asm.spec pacbio.frg

with asm.spec specifying:

unitigger=bogart

overlapper = ovl

ovlErrorRate=0.03

cgwErrorRate=0.10

cnsErrorRate=0.10

utgErrorRate=0.015

utgGraphErrorLimit=0

utgGraphErrorRate=0.015

utgMergeErrorLimit=0

utgMergeErrorRate=0.03

utgErrorLimit = 0

utgBubblePopping = 1

bogBadMateDepth=30

merSize = 14

merylMemory = 128000

ovlStoreMemory = 8192

ovlHashBits = 24

ovlHashBlockLength = 50000000

ovlRefBlockSize = 20000000

To run **PBcR-Celera assembly (CA)** on PacBio reads + Illumina reads + 454 reads:

Version: Celera assembler 8.1

PBcR command and parameters:

For FLX 454 reads use:

sffToCA -output SRRxxx.frg -libraryname SRRxxx -clear discard-n -trim none SRRxxx.sff

For Titanium 454 reads use:

sffToCA -output SRRxxx.frg -libraryname SRRxxx -clear 454 -trim chop SRRxxx.sff

For PacBio read correction with both Illumina and 454 reads:

pacBioToCA -length 100 -partitions 200 –l lib-name -t 16 -s pacbio.spec genomeSize=87000000 -fastq pacbio.fq *.frg

with the same pacbio.spec as used in PBcR-CA assembly on Pacbio reads + Illumina reads.

CA command and parameters are also same as used in PBcR-CA assembly on Pacbio reads + Illumina reads.

To run **Hybrid Celera assembly (CA)** on PacBio reads + Illumina reads + 454 reads:

Version: Celera assembler 8.1

PBcR command and parameters:

Prepare 454 frg files same as in PBcR-CA using Illumina + 454 reads

Use the Illumina corrected PacBio FRG following recipe of PBcR on Pacbio + Illumina

CA command and parameters:

runCA -p ca_hybrid -d ca_hybrid ovlMinLen=300 kickOutNonOvlContigs=1 cgwDemoteRBP=0 cgwMergeMissingThreshold=0.5 -s asm.spec *.frg

with the same pacbio.spec as used in PBcR-CA assembly on Pacbio reads + Illumina reads.

To run **HGAP** assembly on PacBio reads only:

Version: HGAP2

Command and parameters:

source /full/path/to/smrtanalysis/etc/setup.sh

fofnToSmrtpipeInput.py pacbio_h5.list > input.xml

smrtpipe.py --params=settings.xml xml:input.xml

To run **Quiver**:

referenceUploader   -c -n "reference" -p /path/to/HGAP_assembly_folder -f /path/to/HGAP_assembly_folder/data/celera-assembler.scf.fasta --saw="sawriter -blt 8 -welter" --gatkDict="createSequenceDictionary" --samIdx="samtools faidx" -jobId="Anonymous" --ploidy=haploid   --verbose

smrtpipe.py --params=resequencing_settings.xml --output=resequencing/ xml:input.xml
